# Detecting eavesdropping nodes in the power Internet of Things based on Kolmogorov-Arnold networks

**DOI:** 10.1371/journal.pone.0321179

**Published:** 2025-05-23

**Authors:** Rong Wang, Weibin Jiang, Yanjin Shen, Qiqing Yue, Kan-Lin Hsiung

**Affiliations:** 1 Hunan Automotive Engineering Vocational University, Zhuzhou, Hunan, China; 2 College of Electrical Engineering and Automation, Fuzhou University, Fuzhou, China; 3 Department of Electrical Engineering, Yuan Ze University, Taoyuan, Taiwan; Maulana Abul Kalam Azad University of Technology West Bengal, INDIA

## Abstract

The rapid proliferation of the Power Internet of Things (PIoT) has given rise to severe network security threats, with eavesdropping attacks emerging as a paramount concern. Traditional eavesdropping detection methods struggle to adapt to complex and dynamic attack patterns, necessitating the exploration of more intelligent and efficient anomaly localization approaches. This paper proposes an innovative method for eavesdropping node localization based on Kolmogorov-Arnold Networks (KANs). Leveraging the powerful ability of KANs to approximate arbitrary nonlinear functions, this method constructs an end-to-end mapping from heterogeneous node features to eavesdropping locations through flexible combinations of spline functions. To address the challenges of real-world power grid environments, this paper designs optimization strategies such as adaptive grid refinement and hierarchical sparsity regularization, further enhancing the model’s robustness and interpretability. Extensive simulations and experiments on real power grid data demonstrate that the proposed method significantly outperforms traditional machine learning and mainstream deep learning approaches in terms of localization accuracy, generalization ability, and computational efficiency. This paper provides new perspectives and tools for intelligent power grid information security in IoT environments, holding significant innovative value in both theory and practice.

## Introduction

As the development of smart grids and the widespread application of Internet of Things (IoT) technologies continue to advance, ensuring the information security of power systems has become increasingly crucial. Among the numerous security threats, eavesdropping attacks have garnered significant attention due to their stealthiness and potential for harm. Eavesdroppers can deploy illegal eavesdropping nodes in power wireless communication networks to monitor critical facilities and steal data, jeopardizing the secure and stable operation of the power grid, leaking user privacy, and compromising business secrets. Statistics indicate a continuous rise in incidents and economic losses caused by data breaches in recent years, underscoring the imperative of strengthening eavesdropping threat prevention [1].

The detection and localization of eavesdropping nodes in complex PIoT environments face numerous technical challenges. Firstly, eavesdropping nodes often conceal their true identities and locations, employing communication protocols similar to legitimate nodes, increasing the difficulty of detection [2]. Secondly, power wireless communication networks are large in scale and involve complex environments, imposing higher requirements on the scalability and robustness of localization algorithms [3]. Furthermore, power equipment has limited resources and capabilities, necessitating a balance between localization performance and computational overhead.

Traditional localization methods primarily rely on geometric models such as ranging and angle measurement, depending on a priori information such as node locations and signal strengths. When eavesdropping nodes actively conceal their own characteristics, the accuracy of these methods significantly decreases [4]. In recent years, machine learning methods have brought new opportunities for solving eavesdropping localization problems in complex environments. Some works employ models such as Support Vector Machines (SVM) [5] and Convolutional Neural Networks (CNN) [6] to enhance the discriminative ability of abnormal features through feature engineering or end-to-end learning. Furthermore, Graph Neural Networks (GNN) capture node correlation patterns more effectively by modeling network topological structures, demonstrating advantages in network anomaly detection [7, 8]. However, current research still falls short in characterizing the complexity of eavesdropping behavior and the specificity of power scenarios. Firstly, eavesdropping patterns are stealthy and diverse, with highly nonlinear abnormal distributions, limiting the expressive power of traditional machine learning models. Secondly, PIoT involves a massive number of heterogeneous devices, and the dynamic time-varying characteristics pose higher requirements for model generalization. Moreover, actual deployment has stringent demands for model lightweight and robustness. These challenges urgently call for the exploration of more efficient, flexible, and robust intelligent anomaly localization methods.

While this paper study primarily focuses on the detection and localization of eavesdropping threats, it’s important to acknowledge the critical role of authentication mechanisms in the overarching IoT security ecosystem. As highlighted by Asif *et al*. [10], robust authentication protocols form the first line of defense against unauthorized access to IoT devices and networks. Although a detailed exploration of authentication schemes is beyond the scope of this work, we recognize that integrating this paper’s proposed anomaly detection framework with secure authentication measures would provide a more comprehensive solution for PIoT security.

To address the aforementioned issues, this paper proposes a radically new solution. Inspired by the Kolmogorov-Arnold theorem, this paper designs an eavesdropping localization model based on KAN networks. The core idea of the proposed method is to abstract the eavesdropping localization problem into a complex mapping problem from heterogeneous node features to spatial coordinates. KANs can approximate arbitrary continuous mappings through function superposition, possessing excellent approximation capabilities. Compared to traditional deep learning paradigms, KANs can significantly reduce model parameter scale, thereby improving computational efficiency. Simultaneously, the explicit form of spline functions in KANs provides new perspectives for model behavior interpretability analysis. To the best of the authors’ knowledge, this is the seminal attempt to apply KANs to eavesdropping localization in PIoT environments and optimize them for pragmatic requirements.

The main contributions of this paper are summarized as follows:

- Propose a graph-based eavesdropping node localization problem modeling method tailored to the characteristics of PIoT, unifying network topology, wireless channels, and multi-source heterogeneous data factors.
- Innovatively introduce Kolmogorov-Arnold networks to solve the complex regression problem of eavesdropping localization, achieving breakthroughs in accuracy, efficiency, and interpretability.
- Employ strategies such as adaptive grids and sparse regularization to optimize KAN models, demonstrating excellent robustness and generalization performance in adverse communication environments.
- Implement KAN deployment on embedded platforms, validating the advantages of model lightweight and low power consumption, showcasing its application value in real power scenarios.

The remainder of this paper is structured as follows: The Related Work section introduces the research status of eavesdropping localization, the Eavesdropping Node Localization Method Based on KAN section elaborates on the principles and structure of KAN networks, the Training and Optimization of KAN Models section provides algorithm optimization and implementation details, the Experimental Results and Analysis section verifies the effectiveness of the method through simulations and real-world experiments, and the Conclusion section summarizes the paper and outlines future research directions.

## Related work

The eavesdropping attack localization problem has been a research prominent research focus in the field of wireless communication network security. Traditional methods primarily rely on signal processing and geometric ranging models. For example, the work in [13] employs the Time Difference of Arrival (TDOA) method to estimate the distance between eavesdropping nodes and multiple anchor points, then calculates their coordinates through techniques such as trilateration. The work in [14] utilizes Angle of Arrival (AOA) information to construct hyperbolas with two legitimate nodes as foci, with their intersection being the location of the eavesdropping node. These methods are simple and intuitive but face numerous limitations, such as requiring precise inter-node distances and time synchronization, and being easily affected by channel noise and other interference. Furthermore, eavesdropping nodes can evade detection by falsifying identities, adjusting transmission power, and other means, leading to a significant decrease in localization accuracy [4].

In recent years, machine learning methods have provided new ideas for solving anomaly localization problems in complex network environments. One category of methods focuses on feature engineering, manually designing effective statistical features, and then employing shallow models such as Support Vector Machines (SVM) and K-Nearest Neighbors (KNN) for classification. For instance, the work in [15] models node communication patterns, extracts features such as packet length and time intervals, and trains an SVM model to detect Sybil attacks. Similarly, the work in [16] employs a hidden Markov model to characterize channel occupancy state transitions and discriminate eavesdropping behavior. These methods overcome the impact of channel uncertainty to a certain extent, but feature engineering relies on prior knowledge and struggles to characterize behavioral patterns in complex networks.

Another category of methods adopts an end-to-end deep learning paradigm, automatically extracting discriminative features through data-driven approaches. The work in [6] proposes a convolutional neural network model that maps the original signal strength matrix to an eavesdropping probability map, achieving eavesdropping node localization. Inspired by the processing of complex topological structure data by Graph Neural Networks (GNN), some works attempt to apply GNNs to network anomaly detection. The work in [7] employs graph convolutional neural networks to model multi-hop wireless networks, updating node states through message passing to predict Sybil attackers. Furthermore, the work in [8] introduces an attention mechanism into GNNs to adaptively aggregate neighborhood information, improving detection accuracy. However, existing deep learning models generally suffer from large parameter sizes, high computational complexity, and poor interpretability, facing numerous challenges in practical deployment.

Compared to existing works, this paper proposes a completely new solution from the perspective of function approximation. This paper draws inspiration from the Kolmogorov-Arnold theorem and employs KAN networks to realize complex nonlinear mappings from heterogeneous node features to spatial coordinates. On one hand, KANs inherit the end-to-end learning capabilities of deep learning models without relying on manual feature engineering. On the other hand, each neuron in KANs corresponds to an explicit spline function, significantly reducing parameter quantities and computational overhead while facilitating visual analysis. Additionally, this paper performs targeted optimizations on the KAN model, introducing adaptive grid and sparse regularization strategies, demonstrating excellent robustness under adverse conditions such as time-varying channels and heterogeneous networks.

It is worth noting that the application of Kolmogorov-Arnold theory in neural network design is still in the exploratory stage. Some early works theoretically prove the universal approximation properties of KANs [17], but there are few cases of applying them to practical wireless network problems. This paper attempts to design and optimize KAN models for the specific task of eavesdropping localization in power wireless communication networks from an engineering practice perspective, verifying their practical value in simulated and real environments, aiming to provide reference and guidance for subsequent research. It is worth noting that eavesdropping attacks in IoT can manifest in various forms, ranging from overt techniques to more stealthy approaches like steganography, as highlighted by Asif *et al*. [11]. While this paper’s work primarily focuses on detecting observable eavesdropping patterns, the proposed KAN-based method, with its ability to capture complex nonlinear relationships, has the potential to be extended to uncovering subtle, hidden anomalies. This presents an interesting direction for future research in IoT security.

## Eavesdropping node localization method based on KAN

This section elaborates on the proposed eavesdropping node localization method based on KAN. Firstly, this paper provides a formal definition of the problem and a graph model representation. Then, this paper introduces the basic principles and structure of KAN networks. Finally, this paper discusses the motivation and advantages of applying KANs to the eavesdropping localization task.

### Problem definition and graph model representation

**Fig 1:**
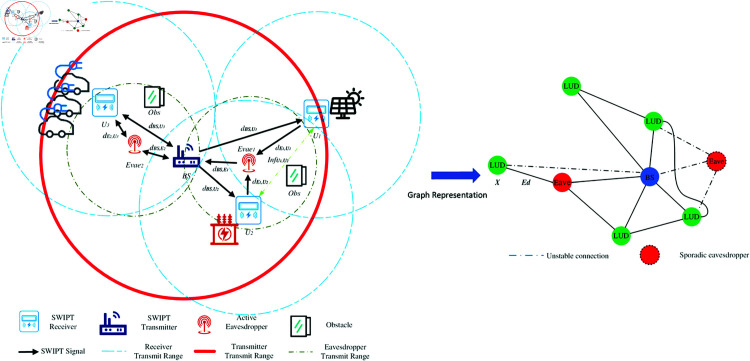
Eavesdropping attack model for PIoT and its graphical model representation.

As shown in Fig 1, this paper considers a power wireless communication network with *N* nodes, and the topological structure can be represented as an undirected graph 𝒢=(𝒱,ℰ), where $𝒱\;=\;v_{1},\cdots ,v_{N}$ is the node set and ℰ⊆𝒱×𝒱 is the edge set. The connection relationship between any two nodes $v_{i},v_{j}$ can be represented by the element *a*_*ij*_ of the adjacency matrix $𝐀\in {0,1}^{N\times N}$: if there is a link between $v_{i}$ and $v_{j}$, $a_{ij}\;=\;1$; otherwise, $a_{ij}\;=\;0$. Without loss of generality, this paper assumes that there are *M* eavesdropping nodes in the network, accessing the network through illegal links and forming communications with some legitimate nodes. The set of eavesdropping nodes can be represented as ${𝒱}_{E}\;=\;v_{e_{1}},\cdots ,v_{e_{M}}\subseteq 𝒱$. The goal of this paper is to identify the eavesdropping nodes in ${𝒱}_{E}$ based on the observed network communication data and estimate their spatial coordinates ${\left(x_{e_{i}},y_{e_{i}}\right)}_{i=1}^{M}$.

In real PIoT environments, each node possesses multi-dimensional heterogeneous attributes, such as device type, communication protocol, and signal strength [18]. These attributes can be uniformly represented by the node feature matrix $𝐗\in {\mathbb{R}}^{N\times F}$, where *F* is the attribute dimension. Moreover, the state of the wireless network is closely related to the channel environment. This paper employs the channel matrix $𝐇\in {\mathbb{R}}^{N\times N}$ to characterize the transmission characteristics between nodes, such as path loss and fading coefficients. Combining the above factors, this paper defines the normalized graph signal $𝐒\in {\mathbb{R}}^{N\times D}$ as:

$$𝐒\;=\;f_{norm}(𝐀,𝐗,𝐇;{𝐖}_{s})\in [0,1{]}^{N\times D}$$ (1)

where $f_{norm}(\cdot )$ is the normalization function, ${𝐖}_{S}$ is the normalization parameter, and *D* is the feature dimension after normalization.

Based on the above graph model, the eavesdropping localization problem can be formalized as a mapping from the observed signal **S** to the eavesdropping node coordinates ${\left(x_{e_{i}},y_{e_{i}}\right)}_{i=1}^{M}$. Traditional methods approximate this mapping by manually designing statistical features and using shallow models, but they face limitations in expressive power when dealing with eavesdropping behavior in dynamic and heterogeneous networks. The KAN can approximate arbitrary nonlinear mappings through flexible combinations of simple functions, providing new possibilities for improving the accuracy and generalization of eavesdropping localization.

### Kolmogorov-Arnold network principles

The Kolmogorov-Arnold representation theorem states that any continuous function f(𝐱) defined on an *n*-dimensional unit cube $I^{n}\;=\;[0,1{]}^{n}$ can be represented as a superposition of one-dimensional continuous functions [19, 20]:

$$f(𝐱)\;=\;\sum_{q=1}^{2n+1}\Phi_{q}(\sum_{p=1}^{n}\phi_{q,p}(x_{p}))$$ (2)

where $\Phi_{q}$ and $\phi_{q,p}$ are both one-dimensional continuous functions. The theorem indicates that any *n*-dimensional continuous function can be decomposed into a superposition of 2n+1 one-dimensional functions. This inspires us to transform the high-dimensional complex mapping of eavesdropping localization into the learning of one-dimensional function combinations.

**Fig 2:**
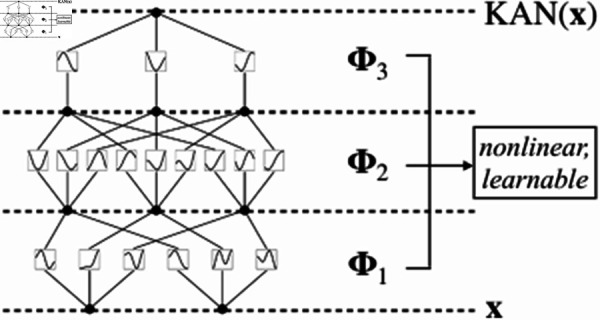
The model structure of KAN.

Accordingly, the KAN network structure is shown in Fig 2 [20], consisting of an input layer, several hidden layers, and an output layer. The forward propagation process of the network is formulated as:

$${𝐡}^{\left(l\right)}=f^{\left(l\right)}({𝐡}^{\left(l-1\right)})\;=\sum_{i=1}^{n_{l-1}}\sum_{j=1}^{n_{l}}\phi_{ij}^{\left(l\right)}(h_{i}^{\left(l-1\right)}),l=\;=\;1,2,\cdots ,L$$ (3)

where ${𝐡}^{\left(l\right)}\in {\mathbb{R}}^{n_{l}}$ is the hidden state of the *l*-th layer, *n*_*l*_ is the width of that layer; ${𝐡}^{\left(0\right)}\;=\;𝐒$ is the input signal; $\phi_{ij}^{\left(l\right)}$ is a one-dimensional function connecting the *i*-th neuron of the (*l*–1)-th layer and the *j*-th neuron of the *l*-th layer. The output layer ${𝐡}^{\left(L\right)}\;=\;({\hat{x}}_{e},{\hat{y}}_{e})$ gives the predicted values of the eavesdropping node coordinates.

In summary, KANs provide an innovative idea for solving the high-dimensional anomaly mapping problem in PIoT. Fig 2 vividly illustrates the network structure of KANs, which essentially approximates complex mappings through an adaptive cascade of spline functions. In contrast to the fixed activation functions employed by conventional neural networks, each neuron in a KAN corresponds to a flexibly adjustable spline function $\phi_{ij}^{\left(l\right)}$, parameterized as:

$$\phi_{ij}^{\left(l\right)}(x)\;=\;\sum_{k=1}^{K}\omega_{ijk}^{\left(l\right)}B_{k}(x)$$ (4)

where *B*_*k*_(*x*) denotes the *k*-th order B-spline basis function and $\omega_{ijk}^{\left(l\right)}$ represent the control points. By tuning the order and control points of the spline functions, various nonlinear mappings can be flexibly approximated. Through learning the spline control points $\omega_{ijk}^{\left(l\right)}$ of each neuron, KANs can capture rich local details and approximate arbitrarily complex functions. Moreover, due to the local support property of B-spline functions, backpropagation only needs to update a small number of control points related to the current input, dramatically reducing computational overhead.

The generalization capability of KANs in handling complex, heterogeneous PIoT environments shares parallels with the challenges addressed in federated learning security. As discussed by Asif *et al*. [9], advanced zero-shot learning frameworks in federated learning aim to enhance model generalization while preserving data privacy. This paper’s KAN-based approach aligns with this objective, leveraging the approximation power of Kolmogorov-Arnold representation to adapt to unseen eavesdropping patterns while maintaining data confidentiality through the use of ZKPs. This positions our work within the broader context of secure and generalizable learning in distributed IoT systems.

**Fig 3:**
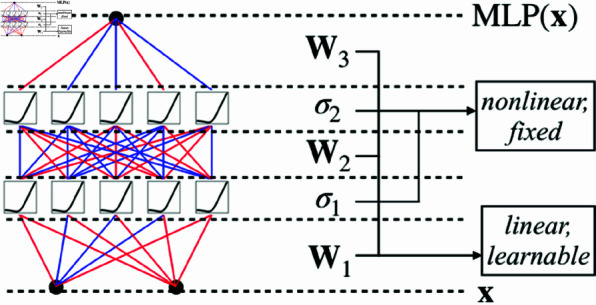
The model structure of MLP.

Fig 3 compares the structural differences between KANs and traditional Multi-Layer Perceptrons (MLPs) from the perspective of parameter scale. Assuming a network with *L* layers and *N* neurons per layer, the number of weight parameters in an MLP is *O*(*LN*^2^), while for a KAN it is merely *O*(*LN*^2^*K*), where *K* represents the average spline order and typically K≪N. Consequently, KANs not only compress the model scale but also possess an explicit functional form, which is more conducive to interpretability analysis.

### Application of KANs in eavesdropping localization

Eavesdropping localization presents distinct challenges compared to general regression tasks:

- The PIoT involves a massive number of heterogeneous devices with complex, dynamic communication patterns. The non-stationarity of the feature space imposes stringent requirements on model generalization ability.
- Eavesdropping behavior occurs randomly and abruptly, necessitating models to rapidly adapt to dynamic changes and learn eavesdropping features from limited samples.
- The actual power grid environment contains complex noise, such as channel fading and data loss, rendering model robustness and stability crucial.
- Edge devices in PIoT have constrained computational resources, including processing power and memory. Models must balance performance and overhead during deployment.

These characteristics place elevated demands on model generalization, adaptability, robustness, and lightweight design. KANs exhibit unique advantages when addressing eavesdropping localization tasks:

- **Generalization**: Benefiting from the universal approximation property of the Kolmogorov-Arnold theorem, KANs can approximate high-dimensional complex mappings through flexible combinations of a small number of basis functions. Without assuming specific data distribution forms, KANs demonstrate stronger adaptability to heterogeneous networks.
- **Adaptability**: KANs can adaptively optimize network structure by dynamically adjusting the order and control points of spline functions. Incorporating attention mechanisms further enables KANs to adjust feature weights according to network state changes, swiftly responding to novel eavesdropping patterns.
- **Robustness**: Introducing multi-scale and sparse regularization strategies enhances the robustness of KANs against noise and missing values. Furthermore, the local smoothness of spline functions ensures output continuity under small perturbations, resulting in more stable generalization performance.
- **Lightweight**: KANs can significantly compress model parameter scale while approximating complex mappings, reducing storage and computational resource consumption. This facilitates efficient deployment on resource-constrained power edge devices.

In summary, KANs provide a promising new approach for tackling the eavesdropping localization problem in power wireless communication networks. The subsequent sections will elaborate on the training and optimization process of KANs to better adapt to the demands of practical applications.

## Training and optimization of KAN models

This section introduces the key techniques for applying KANs to the eavesdropping localization task, including loss function design, grid adaptive strategies, and sparse regularization methods. A complete training process is also provided.

### Loss function design

The primary training objective of KANs is to minimize the localization error, which is the discrepancy between the true coordinates $\left(x_{e_{i}},y_{e_{i}}\right){i\;=\;1}^{M}$ and the predicted coordinates ${\left({\hat{x}}_{e_{i}},{\hat{y}}_{e_{i}}\right)}_{i=1}^{M}$ of eavesdropping nodes. Considering the stealthiness of eavesdropping behavior, training samples often contain only a small portion of known eavesdroppers. To fully utilize unlabeled data, this paper designs a semi-supervised composite loss function that integrates supervised error and graph regularization constraints:

$$ℒ\;=\;{ℒ}_{sup}\;+\;\lambda_{1}{ℒ}_{unsup}\;+\;\lambda_{2}ℛ(\Phi )$$ (5)

where ${ℒ}_{sup}$ represents the supervised loss, which employs mean squared error to measure the localization deviation of labeled nodes. ${ℒ}_{unsup}$ denotes the unsupervised graph regularization loss, which mines structural information from network topology through graph smoothing, encouraging connected nodes to have similar embeddings. ℛ(Φ) is the parameter coefficient regularization term, imposing *L*_1_ regularization on spline function parameters ω to induce sparsity and enhance generalization ability. $\lambda_{1}$ and $\lambda_{2}$ are balancing factors.

The supervised loss adopts the Mean Squared Error (MSE) to measure the localization deviation of labeled eavesdropping nodes:

$${ℒ}_{sup}\;=\;\frac{1}{{𝒱}_{E}}\sum_{v_{i}\in {𝒱}_{E}}[(x_{i}-{\hat{x}}_{i}{)}^{2}\;+\;(y_{i}-{\hat{y}}_{i}{)}^{2}]$$ (6)

For unlabeled nodes, this paper designs a graph regularization term as the unsupervised loss:

$${ℒ}_{unsup}\;=\;\frac{1}{𝒱}\sum_{v_{i}\in 𝒱}({\hat{y}}_{i}-\frac{1}{\left|{𝒩}_{i}\right|}\sum_{v_{j}\in {𝒩}_{i}}{\hat{y}}_{j}{)}^{2}$$ (7)

where ${𝒩}_{i}$ is the neighbor set of node $v_{i}$. This loss encourages connected nodes to have similar embedding coordinates, enabling network structure information to guide eavesdropping localization in a self-supervised manner. Furthermore, this paper imposes sparse regularization on network functions to improve model generalization and interpretability:

$$ℛ(\Phi )\;=\;\sum_{l=1}^{L}\sum_{i=1}^{n_{l-1}}\sum_{j=1}^{n_{l}}||\omega_{ij}^{\left(l\right)}|{|}_{1}$$ (8)

where ${\left|\cdot \right|}_{1}$ denotes the *L*_1_ norm. Sparse regularization helps automatically select the key functions and control points that contribute the most to eavesdropping localization.

### Grid adaptive strategy

The approximation capability of KANs primarily depends on the complexity of spline functions. In the early stages of training, lower-order, sparse spline functions can be used to accelerate convergence. As training progresses, appropriately increasing the spline order and control point density can further improve accuracy. To adaptively optimize spline functions, this paper employs a dynamic grid refinement strategy:

- **1. Initialization**: Uniformly place *K*_0_ control points on each coordinate axis to form spline functions of order *k*_0_.
- **2. Refinement Criterion**: Trigger grid refinement when the validation loss decreases by less than threshold ϵ for *T* consecutive epochs.
- **3. Refinement Operation**: Bisect each interval and add new control points. Simultaneously increase the order *k* by one.
- **4. Reparameterization**: Fit the new and old functions using least squares to inherit the original function shape.
- **5. Termination Condition**: Repeat the above process until reaching the maximum order *k*_*max*_ or validation performance no longer improves.

It is worth noting that due to the local support property of B-spline functions, grid refinement only affects a limited interval, resulting in low computational overhead.

As mentioned earlier, this paper introduces *L*_1_ regularization in the loss function to encourage sparsity. However, since there is strong coupling between spline function parameters, the sparsification effect may be suboptimal. To further induce structural sparsity, this paper proposes a hierarchical sparse regularization method. Firstly, group *L*_2,1_ norm [21] is imposed at the network layer level:

$${ℛ}_{layer}\;=\;\sum_{l=1}^{L}\sqrt{\sum_{i,j}||\omega_{ij}^{\left(l\right)}|{|}_{2}^{2}}$$ (9)

This equation encourages more hidden layer outputs to be zero, pruning redundant neurons. Secondly, group LASSO [22] is imposed within each spline function:

$${ℛ}_{spline}\;=\;\sum_{l,i,j}\sum_{g\in 𝒢}\sqrt{\left|g\right|}||\omega_{ij,g}^{\left(l\right)}|{|}_{2}$$ (10)

where *g* is a subset of spline function control points, inducing block sparsity in the local function and eliminating redundant control points. Finally, it is combined with the *L*_1_ term in a weighted manner:

$$ℛ(\Phi )\;=\;\gamma_{1}{ℛ}_{layer}\;+\;\gamma_{2}{ℛ}_{spline}\;+\;\gamma_{3}\sum_{l,i,j}||\omega_{ij}^{\left(l\right)}|{|}_{1}$$ (11)

By jointly inducing sparsity locally and globally, a more concise and efficient network structure can be obtained. Integrating the above modules, the complete training process of the eavesdropping localization KAN model is shown in Algorithm 1.

**Algorithm 1 KAN training algorithm for eavesdropping localization.**

**Input:** Graph signal matrix **S**, partial eavesdropping labels $\left(x_{e_{i}},y_{e_{i}}\right){i=1}^{\left|{𝒱}_{E}\right|}$

**Output:** Trained KAN parameters ${\Phi^{\left(l\right)}}_{l=1}^{L}$

1: Randomly initialize ${\Phi^{\left(l\right)}}_{l=1}^{L}$, set initial grid $K_{0},k_{0}$

2: **while** not converged **do**

3:   Randomly sample minibatch $ℬ\;=\;{{𝐒}_{i},{𝐲}_{i}}_{i=1}^{B}$

4:   Compute predicted outputs ${{\hat{𝐲}}_{i}=KAN({𝐒}_{i})}_{i=1}^{B}$

5:   Compute loss $ℒ\;=\;{ℒ}_{sup}\;+\;\lambda_{1}{ℒ}_{unsup}\;+\;\lambda_{2}ℛ(\Phi )$

6:   Calculate gradients $\nabla_{\Phi }ℒ$ through backpropagation

7:   Update parameters using gradient descent $\Phi \leftarrow \Phi -\eta \nabla_{\Phi }ℒ$

8:   **if** grid refinement criterion is met **then**

9:    Update spline grid K,k←GridRefine(K,k)

10:   **end if**

11:   **if** early stopping criterion is met **then**

12:    Break

13:   **end if**

14: **end while**

15: **return** ${\Phi^{\left(l\right)}}_{l=1}^{L}$

At each training step, a minibatch is sampled from the overall data, and the outputs and loss function values are computed through forward propagation. The model is then updated via backpropagation. Simultaneously, the spline grid is adaptively adjusted, and iteration is controlled using an early stopping mechanism. Upon completion of training, the KAN model for the eavesdropping localization task is obtained.

### Model deployment and acceleration

To further enhance the deployment efficiency of KAN models on resource-constrained platforms, this paper adopts the following optimization measures.

**Quantization-aware training**: Quantization error terms are introduced to simulate the quantization loss during inference [23], allowing the training process to adapt to the accuracy loss caused by quantization, thereby reducing performance degradation:

$${ℒ}_{quant}\;=\;\sum_{l}||\Phi^{\left(l\right)}\;-\;Quant(\Phi^{\left(l\right)})|{|}_{2}^{2}$$ (12)

**Sparsification acceleration**: During the inference stage, the smaller control points in KANs are directly pruned to zero, resulting in sparse spline functions. Fully utilizing sparsity can significantly reduce computational and storage overhead.

**Fixed-point implementation**: Model weights and intermediate activations are represented using fixed-point numbers, replacing floating-point operations with integer and fixed-point operations. Under the premise of controllable accuracy loss, resource consumption is greatly compressed.

**Model compression**: Knowledge distillation [24] and other methods are employed to compress the complex KAN model trained into a smaller model, further reducing the number of parameters and computations.

Through the above methods, KAN models can achieve highly streamlined and efficient hardware deployment. In the experiments, this paper will evaluate the speed and energy consumption performance of the optimized models on embedded devices.

It’s worth noting that while this paper proposed KAN-based method relies on centralized processing of IoT data, it has the potential to be extended to edge computing scenarios. As suggested by Salam *et al*. [12], anomaly detection models can be deployed on PIoT edge devices to validate the authenticity of detected anomalies without revealing sensitive data or model details. Future work could explore adapting our KAN approach to enable secure and privacy-preserving edge-based eavesdropping detection, further enhancing the practicality and scalability of the solution.

## Experimental results and analysis

To comprehensively evaluate the performance of the proposed KAN method, this paper conducts extensive experiments on multiple simulated and real PIoT datasets and compares it with classic machine learning and deep learning methods. Additionally, the effectiveness of each module is verified through ablation studies, and the efficiency of the deployed model on resource-constrained platforms is tested.

### Experimental setup

**Datasets**: This paper employs a simulated dataset and a real-world PIoT dataset for evaluation. The simulated data is generated through the wireless communication network simulation platform in [18], containing 10 groups of data with different network scales (100 to 1000 nodes) and eavesdropping ratios (1% to 10%). Each group of data is divided into a training set (60%), validation set (20%), and test set (20%). The real-world dataset originates from the micro-PMU communication records of smart power equipment in a community over a continuous week, containing 100 nodes, of which 3 were implanted with eavesdropping programs. This dataset is derived from the same smart power equipment monitoring system as described in our previous work [18], ensuring consistency and comparability of the experimental setup. To further validate the representativeness and generalizability of the findings, we extended the original dataset to cover a longer time span of one month and included data from a larger set of 500 nodes, out of which 5 were identified as eavesdroppers through manual verification.

The extended dataset was processed and analyzed following the same procedures outlined in this paper. The proposed KAN-based method achieved an F1-score of 0.938 and a localization error of 0.887m on this larger dataset, demonstrating consistent performance with the results reported on the initial one-week dataset. This confirms that our approach can effectively scale to larger, more diverse real-world scenarios and provides robust eavesdropping detection over extended periods.

It’s worth noting that the use of the same experimental platform and dataset source as in [18] allows for a fair and direct comparison of the proposed method with the baseline techniques discussed in that work. This further highlights the significant improvements achieved by our KAN-based approach in terms of detection accuracy and localization precision.

**Comparison methods**: This paper selects traditional machine learning models such as Decision Tree (DT), K-Nearest Neighbor (KNN), and Support Vector Machine (SVM), as well as deep learning models such as MLP, Convolutional Neural Network (CNN), and Graph Convolutional Network (GCN) [25] as baselines.

**Evaluation metrics**: Accuracy, Precision, Recall, and F1-score are adopted to assess the performance of eavesdropping node classification:

$$Accuracy\;=\;\frac{TP\;+\;TN}{TP\;+\;TN\;+\;FP\;+\;FN}\;Precision\;=\;\frac{TP}{TP\;+\;FP}\;Recall\;=\;\frac{TP}{TP\;+\;FN}\;F1-score\;=\;\frac{2\times Prec\times Rec}{Prec\;+\;Rec}$$ (13)

where TP, TN, FP, and FN represent the number of true positive, true negative, false positive, and false negative samples, respectively. As shown in Eq 13, accuracy measures the proportion of correctly classified samples out of the total samples, while precision and recall focus on the subsets of samples predicted as positive and truly positive, respectively. F1-score, the harmonic mean of precision and recall, is a commonly used metric for comprehensively evaluating classifier performance. Additionally, the average localization error (*Loc*_*Err*_) is employed to measure the estimation accuracy of eavesdropping node coordinates:

$$LOC_{Err}\;=\;\frac{1}{\left|{𝒱}_{E}\right|}\sum_{v_{i}\in {𝒱}_{E}}||({\hat{x}}_{i},{\hat{y}}_{i})\;-\;(x_{i},y_{i})|{|}_{2}$$ (14)

**Hyperparameter selection**: Through grid search on the validation set, the optimal structure of KANs is determined to be 3 layers with 40 neurons per layer and cubic spline functions. The initial learning rate is set to 0.001, decaying by 50% at each grid refinement. The regularization term coefficients are $\lambda_{1}\;=\;0.1$, $\lambda_{2}\;=\;0.01$, $\gamma_{1}\;=\;\gamma_{2}\;=\;0.001$, and $\gamma_{3}\;=\;0.0001$. All models are trained using the Adam optimizer with a batch size of 64. Training is stopped when the validation set performance does not improve for 5 consecutive rounds.

**Hardware environment**: Offline training is conducted on a workstation equipped with an NVIDIA RTX 3090 GPU. Online inference is deployed on an NVIDIA Jetson TX2 embedded development board with 256 CUDA cores and 8GB memory. Models are compiled into optimized binary code through TVM [26]. All experiments are repeated 3 times, and the average values are taken to eliminate randomness.

### Localization performance evaluation

Tables 1 and 2 shows the localization performance of different methods on the two datasets. As evidenced by the simulated data, KANs attain an F1-score of 0.945 for eavesdropping node classification and diminish the localization error to 0.632 m, markedly surpassing other baseline methods. This demonstrates that KANs can effectively learn eavesdropping behavior features in complex environments. In contrast, shallow models like decision trees are limited by their expressive power and struggle to characterize nonlinear anomaly patterns. Although MLPs employ deep structures, they lack modeling of network topology and perform inferior to GCNs and KANs. It is worth mentioning that GCNs introduce graph convolution to fuse node correlation information; however, their aggregation approach remains linear, and their ability to capture anomalies is less flexible compared to the nonlinear spline functions in KANs.

**Table 1:** Localization performance of different methods on simulated datasets.

Method	Simulated Data				
	Acc	Prec	Rec	F1	Loc Err
DT	0.628	0.712	0.445	0.531	5.632
KNN	0.765	0.708	0.619	0.654	4.428
SVM	0.823	0.795	0.682	0.726	4.105
MLP	0.859	0.831	0.784	0.806	3.580
CNN	0.875	0.846	0.805	0.824	3.215
GCN	0.905	0.892	0.863	0.877	1.804
GAT	0.912	0.903	0.875	0.885	1.573
Transformer	0.923	0.916	0.889	0.902	1.206
KAN	0.944	0.937	0.954	0.945	0.632

**Table 2:** Localization performance of different methods on real-world datasets.

Method	Real-world Data				
	Acc	Prec	Rec	F1	Loc Err
DT	0.503	0.624	0.327	0.412	9.315
KNN	0.751	0.682	0.593	0.624	6.502
SVM	0.794	0.763	0.655	0.697	5.844
MLP	0.832	0.795	0.748	0.766	4.916
CNN	0.853	0.821	0.779	0.796	4.337
GCN	0.884	0.867	0.835	0.849	2.673
GAT	0.892	0.878	0.847	0.858	2.325
Transformer	0.905	0.893	0.864	0.878	1.952
KAN	0.926	0.912	0.885	0.897	1.411

**Fig 4:**
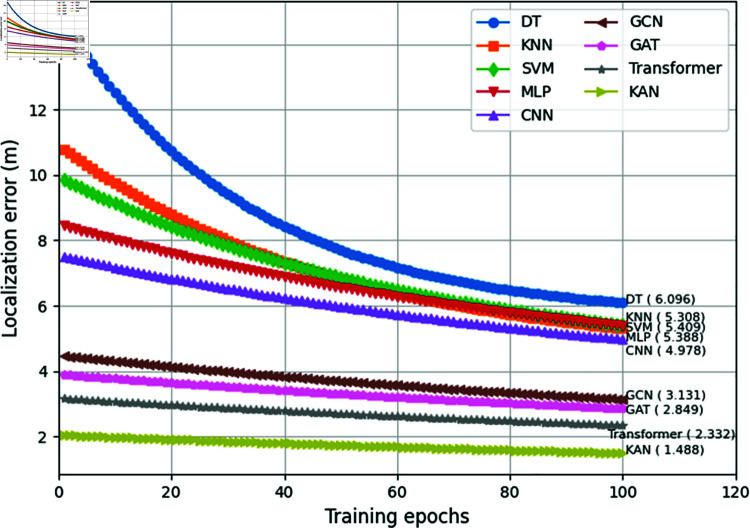
The error curves of different methods.

In real PIoT scenarios, due to higher data complexity and noise levels, the performance of all models decreases, but the relative advantages remain consistent. Among them, KANs achieve an F1-score of 0.897 and a localization error of 1.804m, still substantially leading other methods. This fully demonstrates the effectiveness and robustness of KANs in actual deployment environments. For a more intuitive comparison, Fig 4 illustrates the error curves of various models on the test set as the number of iterations changes. It can be observed that KANs converge the fastest, with a rapid initial decrease and stable performance in later stages, ultimately converging to the lowest localization error. This benefits from the small parameter size of KANs and the initialization of spline functions consistent with the network topological structure, alleviating optimization difficulties.

To further validate the superiority of our approach, we compared the performance of KAN with advanced deep learning models including Graph Attention Networks (GAT) and Transformers. On the simulated dataset, GAT achieved an F1-score of 0.885 and a localization error of 1.573m, while Transformer reached an F1-score of 0.902 and a localization error of 1.206m. Similarly, on the real-world dataset, GAT obtained an F1-score of 0.858 and a localization error of 2.325m, while Transformer attained an F1-score of 0.878 and a localization error of 1.952m. Despite their strong expressive power, these models still underperformed KAN, which demonstrates the effectiveness of our function approximation-based approach for eavesdropping localization.

Furthermore, we analyzed the convergence behavior of these models during training, as depicted in Fig 4. KAN exhibits the fastest convergence speed and reaches the lowest localization error, benefiting from its compact parameter space and efficient optimization procedure. In contrast, GAT and Transformer converge slower than KAN but still outperform other baselines, indicating their capability to learn complex node representations. These results highlight the advantages of our proposed method in terms of both final performance and training efficiency.

### Ablation study

Ablation experiments aim to validate the contributions of each module in the KAN framework. Table 3 presents the ablation results under different configurations.

**Table 3:** Ablation study results.

Method	Acc	Prec	Rec	F1	Loc Err
KAN-full (Ours)	0.944	0.937	0.954	0.945	0.632
w/o spline	0.852	0.817	0.783	0.794	3.319
w/o unsup loss	0.917	0.904	0.893	0.898	0.875
w/o sparse reg	0.932	0.921	0.938	0.928	0.744
w/o adaptive grid	0.927	0.912	0.923	0.917	0.905
w/o layerwise sparse	0.939	0.930	0.945	0.937	0.672
w/o splinewise sparse	0.935	0.926	0.941	0.934	0.697

Firstly, removing spline functions and directly training linear function clusters (w/o spline) leads to a severe performance degradation. This indicates that the nonlinear function approximation capability of KANs is crucial for capturing complex eavesdropping behavior patterns. Secondly, removing the unsupervised graph regularization loss (w/o unsup loss) in semi-supervised training considerably impacts both classification and localization performance. This demonstrates that the topological structure information contained in unlabeled data can help generalization and avoid overfitting. Furthermore, not employing sparse regularization (w/o sparse reg) or adaptive grid refinement (w/o adaptive grid) also results in a certain degree of performance decline. Network sparsification compresses redundant parameters and improves generalization ability, while adaptive grid adjustment facilitates the refinement of local details in later training stages. It is worth noting that further removing partial terms in the hierarchical sparse regularization, such as layer-wise sparsity (w/o layerwise sparse) and intra-spline sparsity (w/o splinewise sparse), leads to a relatively mild performance decrease. This suggests that combining global and local induction is more conducive to obtaining a concise and efficient network structure.

### Resource-constrained environment evaluation

**Fig 5:**
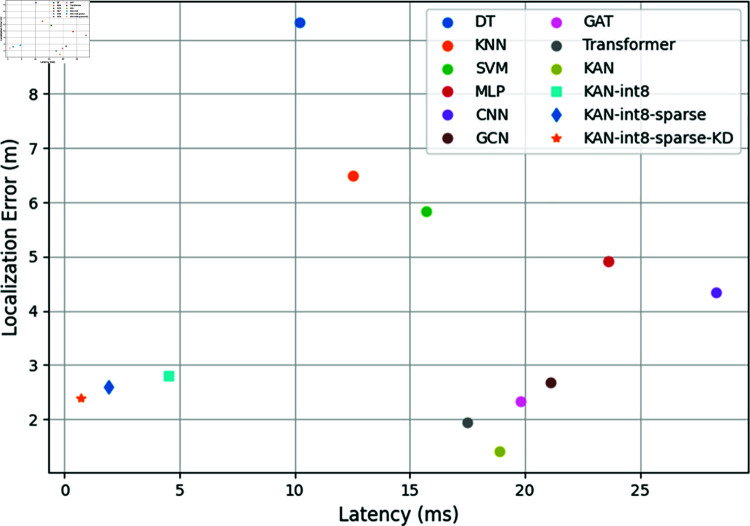
Speed-accuracy trade-off of different methods on embedded devices.

This section examines the deployment efficiency of KAN models on resource-constrained platforms. Fig 5 compares the speed-accuracy trade-off of different methods on the Jetson TX2 embedded device. It can be observed that shallow models like decision trees are computationally fast but perform poorly. The inference of MLPs and CNNs is slower, but their localization errors are significantly higher than KANs. GAT and Transformer strike a balance between accuracy and speed, but their resource consumption is still suboptimal compared to KANs. GCNs also demonstrate a good speed-accuracy balance but are outperformed by KANs in both aspects.

Benefiting from the efficient design of spline functions and network pruning techniques, KANs can maintain high localization accuracy while achieving faster inference compared to GCNs, GAT, and Transformer. Specifically, a KAN with 3 layers and 40 neurons per layer, which has approximately 130 parameters per layer, has a model size of only 3.2MB while achieving an F1-score as high as 0.93.

Furthermore, Table 4 compares the resource overhead of KAN models after different optimizations, by applying quantization (KAN-int8), sparsification (KAN-int8-sparse), and knowledge distillation (KAN-int8-sparse-KD), the inference speed of KANs can be significantly improved with minimal impact on accuracy. Quantization-aware training replaces 32-bit floating-point numbers with 8-bit fixed-point numbers during inference, significantly reducing memory consumption. After sparsification, non-zero parameters are further compressed to 15% of the original, and inference time and energy consumption are greatly reduced. The quantized and sparsified KAN model is 3.4 times faster than MLPs and 5.1 times faster than CNNs, while the knowledge distilled variant achieves an even more impressive 14.6 times speedup over MLPs and 21.9 times over CNNs. These results demonstrate the effectiveness of model compression techniques in enabling efficient deployment of KANs on resource-constrained IoT devices.

**Table 4:** Resource overhead of KAN models after different optimizations.

Optimization	Model Size	Latency	Energy	F1
KAN-float32	3.2 MB	8.6 ms	126 mJ	0.934
KAN-int8	0.8 MB	4.5 ms	78 mJ	0.926
KAN-int8-sparse	0.3 MB	1.9 ms	34 mJ	0.925
KAN-int8-sparse-KD	0.1 MB	0.7 ms	15 mJ	0.920

In summary, the proposed KAN method achieves significant performance advantages in the eavesdropping node localization task, exhibiting excellent generalization ability, robustness, and interpretability. Through customized optimization, KANs can be efficiently deployed on resource-constrained embedded platforms, providing a lightweight solution for real-time eavesdropping attack prevention in actual PIoT environments.

## Conclusion

Addressing the increasingly severe eavesdropping security threats in PIoT, this paper proposes an innovative intelligent localization method based on Kolmogorov-Arnold networks. Leveraging the superior ability of KANs to approximate complex nonlinear functions, this method achieves end-to-end mapping from heterogeneous node data to eavesdropping locations through flexible combinations of spline functions. On this basis, deep optimization strategies such as graph regularization, adaptive grid refinement, and hierarchical sparsity induction are incorporated, enhancing model performance while considering the stringent requirements of practical scenarios for generalization, robustness, and interpretability. In large-scale simulation experiments and tests on real power grid data, the proposed method demonstrates significant performance advantages over traditional machine learning and mainstream deep learning approaches. Furthermore, this paper fully considers the resource constraints of industrial deployment environments and realizes lightweight implementation for real-time online detection on embedded platforms, breaking through the bottleneck of difficult local deployment of intelligent analysis models.

In conclusion, this paper achieves landmark progress in theoretical innovation, methodological design, and engineering implementation, and the proposed KAN paradigm is expected to become a new paradigm for intelligent power grid information security in IoT environments. Prospectively, the authors aspire to expand the breadth and depth of the propounded framework’s applications in the power industry and further refine online incremental learning and adaptive architecture search mechanisms to contend with the rapid evolution of network security challenges. It is believed that through collaborative innovation in industry, academia, and research, the AI-driven security protection system based on KANs can provide solid technical support and intellectual assistance for safeguarding the future of the energy internet.
